# Pharmacologic Targeting of Chromatin Modulators As Therapeutics of Acute Myeloid Leukemia

**DOI:** 10.3389/fonc.2017.00241

**Published:** 2017-10-12

**Authors:** Rui Lu, Gang Greg Wang

**Affiliations:** ^1^Lineberger Comprehensive Cancer Center, University of North Carolina at Chapel Hill School of Medicine, Chapel Hill, NC, United States; ^2^Department of Biochemistry and Biophysics, University of North Carolina at Chapel Hill, Chapel Hill, NC, United States

**Keywords:** epigenetic modulator, small-molecule inhibitors, acute myeloid leukemia, bromodomain, MLL, EZH2, DNMT3A, DOT1L

## Abstract

Acute myeloid leukemia (AML), a common hematological cancer of myeloid lineage cells, generally exhibits poor prognosis in the clinic and demands new treatment options. Recently, direct sequencing of samples from human AMLs and pre-leukemic diseases has unveiled their mutational landscapes and significantly advanced the molecular understanding of AML pathogenesis. The newly identified recurrent mutations frequently “hit” genes encoding epigenetic modulators, a wide range of chromatin-modifying enzymes and regulatory factors involved in gene expression regulation, supporting aberration of chromatin structure and epigenetic modification as a main oncogenic mechanism and cancer-initiating event. Increasing body of evidence demonstrates that chromatin modification aberrations underlying the formation of blood cancer can be reversed by pharmacological targeting of the responsible epigenetic modulators, thus providing new mechanism-based treatment strategies. Here, we summarize recent advances in development of small-molecule inhibitors specific to chromatin factors and their potential applications in the treatment of genetically defined AMLs. These compounds selectively inhibit various subclasses of “epigenetic writers” (such as histone methyltransferases MLL/KMT2A, G9A/KMT1C, EZH2/KMT6A, DOT1L/KMT4, and PRMT1), “epigenetic readers” (such as BRD4 and plant homeodomain finger proteins), and “epigenetic erasers” (such as histone demethylases LSD1/KDM1A and JMJD2C/KDM4C). We also discuss about the molecular mechanisms underpinning therapeutic effect of these epigenetic compounds in AML and favor their potential usage for combinational therapy and treatment of pre-leukemia diseases.

## Introduction

Epigenetic modifications, including DNA methylation and a myriad of post-translational modifications of the DNA-packaging histone proteins, represent a fundamental means for regulating gene expression and other DNA-templated processes (1–4). These modifications of DNA or histones are increasingly appreciated to be dynamically regulated by epigenetic modulators, a broad class of proteins that consist of “epigenetic writer” enzymes catalyzing chromatin modification, “epigenetic eraser” enzymes removing the modification, “epigenetic readers” or “effectors” recognizing the modification to elicit biological consequences, and various other cellular regulators that indirectly influence the level or readout of epigenetic modification (2, 5). While the dynamic regulation of epigenetic modification enables cells to adapt and function differently in response to developmental and environmental cues, their mis-regulation often perturbs gene expression and cellular function leading to pathogenesis of human disease such as cancer. Indeed, recent deep sequencing of human cancer patient samples has identified novel recurrent mutations in genes encoding a wide range of epigenetic modulators and even histones themselves (6–9).

Acute myeloid leukemia (AML), a common malignancy of myeloid-lineage precursor cells in the blood, is characterized by two hallmarks, uncontrolled cell proliferation and impaired differentiation. Previously, progression and characteristics of AML were linked to several key pathways (10, 11), including inactivation of tumor suppressors [such as TP53 and *Wilm’s Tumor-1* (WT1)], gain-of-function mutation of oncogenic kinases (such as FLT3, NRAS, and KRAS), and stem cell transcription factors (TFs) [such as rearrangement and/or overexpression of HOX cluster genes and their cofactors such as MEIS1 (12–14)], as well as inactivating mutation of differentiation-promoting TFs (such as PU.1 and CEBP/α). Recently, deep sequencing of samples from human patients with AML and pre-leukemia diseases such as myelodysplastic syndrome (MDS) and clonal hematopoiesis of indeterminate potential (CHIP) additionally revealed frequent somatic mutations of genes involved in epigenetic modulation or RNA splicing (11, 15–26). Among the various affected epigenetic pathway genes include the *DNA (cytosine-5)-methyltransferase 3 A* (DNMT3A, a DNA methylation “writer”), *Tet Methylcytosine dioxygenase 2* (TET2, a DNA methylation “eraser” or demethylase*), Enhancer of zeste homolog 2* [EZH2/KMT6A, a “writer” mediating methylation of histone H3, Lys27 (H3K27)], *Additional Sex Combs Like 1 and 2* (ASXL1 and ASXL2, an EZH2-associated cofactor family), the Cohesin complex (SMC3-SMC1-RAD21-STAG) genes, and *Isocitrate Dehydrogenase 1* and *2* (IDH1 and IDH2). These newly identified somatic mutations of DNA/chromatin modifiers and structural organizers are in agreement with previous karyotyping/FISH-based analyses of AML patients, which already identified recurrent chromosomal translocation or abnormality of genes encoding various members of epigenetic “writers” (MLL/KMT2A, NSD1/KMT3B, NSD3/WHSC1L1/KMT3F) (27–31), “erasers” (JARID1A/KDM5A) (32, 33), and “readers” (PHF23) (32, 34). Importantly, mutations of *DNMT3A, TET2, IDH1/2*, or *ASXL1* were frequently detected among apparently healthy individuals with clonal hematopoiesis or CHIP (22, 24, 35, 36) and in AML patients who received complete disease remission after chemotherapy (26, 35, 37–39), supporting the pivotal roles of epigenetic deregulation in initiation, clonal evolution and relapse of AMLs.

In contrast to significant advances in molecular appreciation of human AML’s mutational landscape and putative “driving” pathways, chemotherapy remains as the frontline treatment for most AML patients, with an exception of all-trans retinoic acid (ATRA) used as targeted therapy of the acute promyelocytic leukemia (APL) subtype. AML patients still suffer from low overall survival and a high rate of recurrence, demanding new treatments to be developed. Recent studies of AML and other tumors have increasingly shown that genetic lesion of epigenetic modulator often induces a subsequent chain reaction leading to aberrations in chromatin modification/remodeling, gene-expression program, and cellular states during tumorigenesis (2, 5, 29, 40–43). Thus, pharmacologic targeting of epigenetic players responsible for the above chromatin/gene mis-regulation shall represent new mechanism-based strategies for therapeutic intervention. This review aims to summarize recent advances in specific inhibition of histone-modifying enzymes and regulatory proteins as potential AML therapeutics, with the already discovered inhibitors sub-grouped into the categories targeting either the “writing,” “reading,” or “erasing” function of epigenetic modulators (Table 1).

**Table 1:** Epigenetic therapies in acute myeloid leukemia (AML): targets, compounds, and clinical development.

Targets	Role in epigenetic regulation	Representative compounds	Indications	Clinical development
**Writers**				

MLL protein complex	H3K4 methyltransferase	MM-401	MLL-rearranged AML	Preclinical
		MIV-6R^a^		
		MI-503^a^		
G9A	H3K9 methyltransferase	UNC0648	HOXA9-overexpressed AML	Preclinical
EZH2	H3K27 methyltransferase	GSK126	MLL-rearranged AML	Preclinical
		UNC1999		
		EPZ005687		
		Tazemetostat		
DOT1L	H3K79 methyltransferase	SGC0946	MLL-rearranged AML, and others	Phase I
		EPZ-5676		
PRMT1	H4R3 methyltransferase	AMI-408	MLL-EEN/GAS7, MOZ-TIF2 and AML1-ETO AML	Preclinical

**Readers**				

Bromodomain proteins	Histone acetylation readers	JQ1	MLL-rearranged AML, and others	Phase I and Phase II
		I-BET151		
		I-BED762		
		CPI-0610 OTX015		
		TEN-01		
		FT-1101		
		GSK525762		
NUP98-PHF23 or NUP98-JARID1A	H3K4me3 readers	Disulfiram	AMLs with NUP98-PHF23 or NUP98-JARID1A	Preclinical

**Erasers**				

Histone deacetylases	Histone deacetylases	Vorinostat	AML	Phase I and Phase II for AML; FDA approved for T cell lymphoma and multiple myeloma
		Romidepsin		
		Panobinostat		
		Givinostat		
		Mocetinostat		
		Ricolinostat		
		AR-42		
		CUDC-907		
LSD1	H3K4 demethylase	GSK2879552	MLL-rearranged AML, and others	Phase I
		ORY-1001		
KDM4C	H3K9 demethylase	SD70	MLL-EEN/GAS7 and MOZ-TIF2 AML	Preclinical

*^a^MLL/Menin inhibitor is likely to act through inhibiting MLL fusion and not wild-type MLL proteins and probably should not be listed among the “writer” inhibitor category*.

## Targeting Chromatin “Writers”

### MLL Inhibitors (MLLi)

The *Mixed-Lineage Leukemia* gene (*MLL/MLL1/KMT2A*) encodes one of the KMT2 family of methyltransferase enzymes that contain multiple structural domains, including a C-terminal SET domain catalyzing methylation of histone H3, Lys4 (H3K4) (44–46). *MLL* rearrangement and translocation, which typically affect one allele, are responsible for about 70% of infant leukemias and 5–10% of childhood and adult AML cases (28, 29). Often, the leukemia-associated *MLL* gene rearrangement produces the MLL fusion oncoprotein that loses MLL’s C-terminal SET domain and gains a partial sequence from its fusion partner such as AF4, AF9, AF10, or ENL, which recruits the DOT1L-associated transcription elongation complexes. MLL fusion oncoproteins still retain MLL’s N-terminal domains, which mediate chromatin association and interaction with functional cofactors such as Menin. Previously, the remaining wild-type *MLL* allele in cancer cells was shown to be critical for leukemogenesis induced by MLL fusion (47); however, a recent study reported that MLL2/KMT2B, another trithorax family methyltransferase that is most closely related to MLL/KMT2A (48), sustains growth of *MLL-*rearranged leukemia and represents a more relevant drug target (49). While the transcription elongation activity acquired by MLL fusion remains as an attractive targeting strategy (see the section of DOT1Li), these studies have justified development of MLL1/2 inhibitors (MLLi) for the treatment of *MLL-*rearranged leukemias.

**Figure 1:**
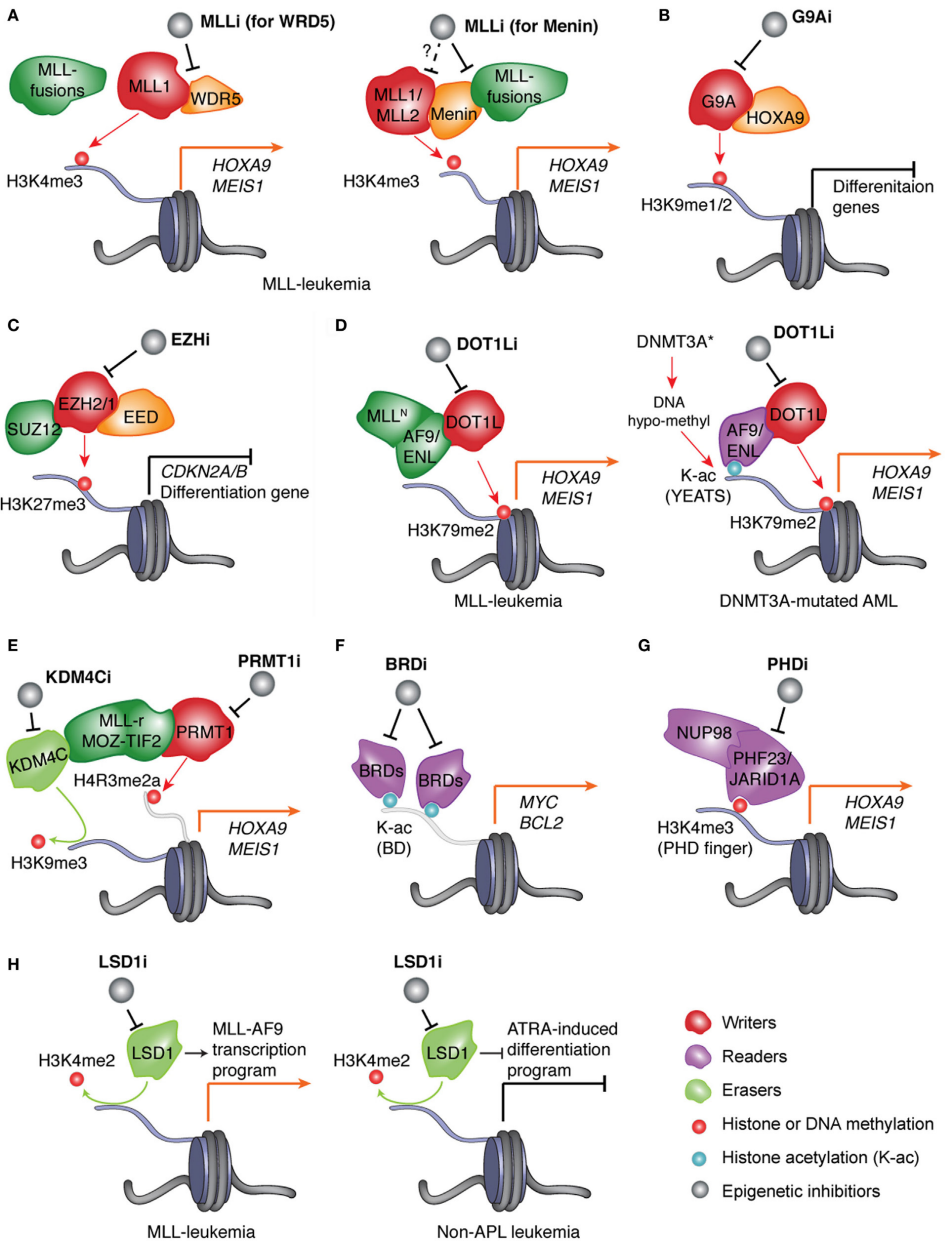
Pharmacological inhibition of the epigenetic “writers,” “readers,” or “erasers” responsible for deregulation of chromatin modification and gene expression in AMLs. **(A)** In leukemias with *MLL* rearrangement (MLL-r), protein complexes assembled by the wild-type MLL and aberrant MLL fusion proteins induce H3K4me3 and H3K79me2, respectively, to cooperatively mediate activation of MLL targets such as “stemness” genes *HOXA9* and *MEIS1*. Inhibitor of MLL (MLLi) disrupts physical association of MLL (MLL1 or MLL2) and MLL-fusion to its interacting partner, either WDR5 (left) or Menin (right), thereby preventing target gene activation and AML development. **(B)** HOXA9, a transcription factor (TF) found overexpressed in ~50–70% of AML patients, promotes leukemogenesis partly through recruiting G9A, an H3K9me1/2-specific “writer” enzyme, to suppress gene-expression programs crucial for myeloid differentiation. Inhibitor of G9A (G9Ai) targets this differentiation-arrest mechanism in AMLs with HOXA9 overexpression. **(C)** In AMLs, treatment with inhibitor of EZH2 and/or EZH1 (EZHi) results in suppression of H3K27me3 and de-repression of polycomb repressive complex 2 (PRC2) target genes, which include tumor suppressor genes (such as CDKN2A/B) and myeloid differentiation-associated genes. **(D)** Left panel: in *MLL*-rearranged leukemias, MLL fusion partners such as AF9 and ENL recruit DOT1L, an H3K79me2-specific “writer” enzyme, to maintain high expression of target genes such as *MEIS1* and *HOXA9*. Right panel: in normal-karyotype AMLs with *DNMT3A* mutation, focal decrease of DNA methylation (i.e., hypo-methylation) results in increase of histone acetylation (K-ac) and binding of the YEAST domain-containing K-ac “reader” proteins AF9 and ENL, which subsequently recruit DOT1L to promote H3K79me2 and transcriptional activation/elongation of “stemness” genes. In both genetically defined AML subtypes, inhibitor of DOT1L (DOT1Li) blocks the above oncogenic program and leukemia progression. **(E)** In leukemias with aberrant fusion of MLL or MOZ-TIF2, PRMT1, an H4R3-specific methyltransferase/“writer,” and KDM4C, an H3K9-specific demethylase/“eraser,” are recruited by leukemic fusion oncoproteins to modulate histone methylation and promote target gene activation. Blockage of PRMT1 or KDM4C provides a new treatment strategy. **(F)** In AMLs, inhibitor of bromodomain (BRD)-containing K-ac “readers” (BRDi) selectively blocks interaction of bromodomain proteins (BRD4 and related BRD2/3) with K-ac and represses expression of vital oncogenes such as *MYC* and *BCL2*, thus suppressing leukemic growth. **(G)** In AML patients, aberrant rearrangement of the gene encoding the H3K4me3-“reading” proteins JARID1A/KDM5A and PHF23 produces the leukemogenic fusion protein NUP98-JARID1A and NUP98-PHF23, respectively, which rely on their H3K4me3-“reading” plant homeodomain (PHD) finger domains to maintain high expression of AML-associated genes. Inhibitor of PHD fingers (PHDi) shall provide an attractive therapeutic method for these AML patients. **(H)** Left: in MLL-rearranged leukemia, inhibitor of LSD1 (LSD1i) downregulates MLL target genes and inhibits leukemia development. Right: in non-acute promyelocytic leukemia (APL) leukemia, LSD1i promotes all-trans retinoic acid (ATRA)-induced cell differentiation thereby suppressing leukemogenesis.

Using the structure-guided design, Cao et al. developed an MLLi termed MM-401 (Figure 1A, left and Table 1) to disrupt direct interaction of MLL1 with WDR5, a cofactor associated with the SET domain of MLL/KMT2 enzymes, and thus inhibit MLL1’s methylase or “writer” function (50). *In vitro* biochemical assays showed that MM-401 specifically targets WDR5 interaction to MLL1, and not other MLL/KMT2 family enzymes. Treatment with MM-401 blocked proliferation and induced myeloid differentiation of *MLL-*rearranged leukemia cells while not significantly affecting normal blood stem/progenitor cells (50). A recent study reported that MLL2 represents a more relevant therapeutic target in a range of *MLL-*rearranged leukemia models and that MLL2 and MLL1 collaborate to maintain oncogenesis *via* regulating distinctive gene-expression pathways (49). Therefore, dual inhibitors of MLL2 and MLL1 or a specific one against MLL2 need to be developed and may provide a more effective treatment strategy.

Menin, a cofactor associated with the N-terminal region of both MLL fusion and wild-type MLL1/2 proteins, is required for MLL- and MLL fusion-mediated target gene activation and for leukemic transformation caused by *MLL* rearrangement (51–55). Menin is required for association and/or recruitment of MLL and MLL fusion proteins to their gene targets and represents a validated drug target of *MLL-*rearranged leukemia. Recently, through high-throughput screening and structure-based development, a series of MLLi, including MIV-6R (56), MI-463, and MI-503 (57), were discovered and optimized to disrupt MLL–Menin interaction, with some achieving *in vitro* inhibition in the nanomole range (Figure 1A, right; Table 1). These MLL–Menin inhibitors efficiently suppressed growth of *MLL-*rearranged leukemia cells *in vitro*/vivo and did not affect that of non-*MLL-*rearranged leukemias. Treatment with these MLLi led to down-regulation of gene-expression programs enforced by MLL fusion, such as *HOXA9* and *MEIS1*, in the leukemia cells. The effect of MLL–Menin inhibitors on steady-state normal hematopoiesis appears to be small (57), suggesting that their anti-leukemia effect is mainly through inhibiting Menin interaction to MLL fusion and not wild-type MLL1 proteins. For this reason, MLL–Menin inhibitors should not be categorized as the “writer” inhibitor. However, it is worthy noting that, besides MLL1/KMT2A, Menin also interacts with MLL2/KMT2B through conserved interfaces (46, 51, 53). It remains to be determined whether the above MLLi also targets MLL2, a recently validated oncoprotein that sustains *MLL-*rearranged leukemias (49). For convenience, we decide to list the MLL–Menin inhibitors as MLLi and “writer” inhibitors (Table 1).

### G9A Inhibitors (G9Ai)

*Euchromatic histone lysine methyltransferase 2* (*EHMT2*, also known as *G9A/KMT1C*) encodes a methyltransferase that catalyzes mono/di-methylation of histone H3, Lys9 (H3K9me1/2), a histone modification correlated with gene silencing. Knockout of *G9A* in hematopoietic systems led to decreased proliferation of myeloid progenitors without affecting the function of long-term repopulating hematopoietic stem cells (58). In mouse AMLs induced by *HOXA9*, a homeodomain TF gene found over-expressed in about 50–70% of human AMLs, loss of *G9A* suppressed leukemogenesis. Mechanistically, G9A physically interacts with HOXA9. Inhibition of G9A led to de-repression of HOXA9 target genes (58). UNC0638 (59), a recently developed G9Ai, demonstrated similar AML therapeutic effect (Figure 1B; Table 1). While no method is currently available for directly targeting HOXA9 oncoprotein, the above studies provide an alternative strategy.

### EZH Inhibitors (EZHi)

EZH2/KMT6A serves as the catalytic subunit of the polycomb repressive complex 2 (PRC2) mediating transcriptional repression through tri-methylation of H3K27 (H3K27me3) (60). EZH1, an EZH2-related methylase, can partially compensate EZH2’s functions on a subset of gene targets when assembled in a separate complex with the same set of PRC2 components such as SUZ12 and EED (60, 61). Genomic deletion and loss-of-function mutations of *EZH2/KMT6A* were frequently found in MDS and other myeloid malignancies (62), whereas its gain-of-function mutations occur in 10–20% of B-cell lymphoma patients (63–65). Such *EZH2/KMT6A* somatic mutation is rare among AMLs (66). Recent investigation of animal blood cancer models, however, has shown that complete loss of *EZH2* promotes MDS development but prevents AML transformation (67). MDS induced by *EZH2* loss requires EZH1 for disease progression (68), indicating a context-dependent role of these PRC2 enzymatic complexes in development of MDS and blood malignancy. Furthermore, several studies demonstrated that the *MLL-*rearranged leukemias require functionality of EZH2 and/or EZH1 to maintain leukemogenecity (69–74). Mechanistically, these PRC2 enzymes suppress genes related to tumor suppression (such as *Cdkn2a/b*) and cell differentiation (such as *Egr1*) through inducing gene-repressive H3K27me3/2 (Figure 1C). Additionally, PRC2 was found to promote expression of MYC-associated gene signatures probably *via* an indirect mechanism. Furthermore, about 5–10% of AML patients carry the inactivating mutation of the *WT1* gene, which was shown to induce a DNA hyper-methylation phenotype through interfering with WT1-mediated recruitment of TET DNA demethylases (75, 76). The induced DNA hyper-methylation sites were found enriched in myeloid differentiation genes and PRC2 targets, and *EZH2* is highly expressed in *WT1-*mutated AMLs to maintain repression of genes with DNA hyper-methylation, leading to cell differentiation block (77). Importantly, in cellular and murine models of *MLL-*rearranged (69, 70, 72) or *WT1-*mutated AMLs (77), knockdown or knockout of PRC2 inhibited cell proliferation and restored gene-expression programs involved in myeloid differentiation. These studies unveiled the oncogenic function of PRC2 and EZH2 in these genetically defined AMLs, supporting PRC2 as a drug target of AML.

Due to frequent overexpression and gain-of-function mutation of *EZH2* in solid cancer and lymphoma, several pharmaceutical companies have embarked on high-throughput screening campaigns leading to discovery of a series of small-molecule compounds (Table 1) that compete binding of S-adenosyl-methionine (SAM), the methyl donor of PRC2, thereby suppressing PRC2’s methyltransferase activity (78–82). These EZHi compounds demonstrate high selectivity and high potency toward EZH2 and/or EZH1. In *MLL-*rearranged AML models, dual inhibition of EZH2 and EZH1 by an EZHi, UNC1999, derepressed PRC2 target genes and significantly suppressed AML malignant growth *in vitro* and *in vivo* (74) (Figure 1C). Treatment of *WT1*-mutated AML cells with GSK126 (79), an EZH2-selective inhibitor, had similar anti-cancer effect (77). Currently, several EZHis show drug-like properties and are used in clinical trials of lymphoma treatment. Their potential therapeutic effect in AMLs remains to be determined in clinical settings.

### DOT1L Inhibitors (DOT1Li)

Disruptor of telomeric silencing 1-like (DOT1L/KMT4) is a histone H3 Lys79 (H3K79)-specific methyltransferase that regulates gene transcriptional elongation, telomeric silencing, and DNA damage response (83). Biochemical interaction studies found that DOT1L interacts with transcriptional elongation factors including AF4, AF9, AF10, and ENL, which are also common fusion partners of *MLL-*rearrangement in AMLs (29, 84–86). DOT1L loss-of-function studies in *MLL-*rearranged leukemias support its crucial role in leukemogeneicity, possibly through maintaining expression of target transcripts of MLL-fusion such as *HOX* cluster genes and *MEIS1* (84, 87–91).

Structure-based design has led to development of several DOT1Li (Table 1) that specifically targets the SAM-binding pocket of DOT1L enzymes (92, 93). Consistent with DOT1L loss-of-function studies, these DOT1Li also selectively inhibited expression of MLL-fusion target genes such as *HOXA9* and *MEIS1* and selectively killed *MLL-*rearranged leukemia cells and xenografted tumors (90–92, 94). Furthermore, recent investigation supports that DOT1L can potentially serve as a therapeutic target of other genetically defined AMLs, which include the subtype with translocation of *NUP98-NSD1* (95), somatic mutation of *DNMT3A* (96, 97), *NPM1* (98) or *IDH1/2* (99), or overexpression of *MN1* (100). While *NUP98-NSD1* induced leukemic transformation through direct targeting and epigenetic modulation of AML-promoting “stemness” genes (*HOX* gene clusters and *MEIS1*) (30), a *DNMT3A* hotspot mutation (DNMT3A^R882H^) was recently found to focally suppress DNA methylation at cis-regulatory elements of these genes thereby promoting their transcription activation (96). In addition, aberrant over-expression of *HOX* cluster genes is a hallmark of AMLs that harbor *NPM1* mutation (98), and overexpression of *MN1* was found to induce an aggressive myeloid leukemia that strictly relies on the same “stemness” genes-expression program in the leukemia-initiating cells (100). Leukemia cells from the above AML subtypes were found generally sensitive to DOT1Li, and DOT1Li treatments repressed “stemness” gene-expression programs, supporting a broader role of DOT1L and “stemness” TF nodes in AML biology (Figure 1D). EPZ-5676 (94) represents the first DOT1Li used for clinical trials for *MLL-*rearranged leukemia; however, drug-like properties of the disclosed DOT1Li such as half-life *in vivo* are generally poor and need to be improved.

### PRMT1 Inhibitors (PRMT1i)

*Protein arginine methyltransferase 1* (*PRMT1*) encodes a methyltransferase for histone H4 arginine-3 (H4R3) and associates with gene activation. PRMT1 was shown to interact with AML1-ETO, a gene fusion product defining AML with t(8;21) translocation, activate the downstream target genes of AML1-ETO, and promote progression of AML1-ETO-asssociated leukemia (101). Recent studies have additionally demonstrated specific requirement of PRMT1 in leukemogenesis induced by *MLL*-rearrangement (such as *MLL-GAS7*) or the *MOZ-TIF2* translocation (102, 103). Similar to what was found in t(8;21) AMLs, PRMT1 physically associates with these leukemic fusion oncoproteins and is required for high expression of their target genes such as *HOX* and *MEIS1*, supporting targeting PRMT1 as new AML therapeutics. Indeed, in various leukemia cell lines and animal models with *MLL* fusion or *MOZ-TIF2*, AMI-408 (104), a PRMT1i, suppressed AML growth (103) (Figure 1E; Table 1). These works have established a foundation for further validation of PRMT1i’s therapeutic effect in clinical settings.

## Targeting Chromatin “Readers”

Epigenetic or chromatin “readers” are a subclass of factors that specifically recognize DNA or histone modification to induce subsequent events and elicit functional readout of the modification (1, 2, 105–107). Compared to a generally high druggability of chromatin-modifying “writer” or “eraser” enzymes, that of various epigenetic “reader” families varies (108). Despite challenges, targeting chromatin “reader” function is increasingly considered as promising partly due to recent success in discovery of bromodomain (BRD) protein inhibitors.

### BRD Inhibitors (BRDi)

BRD-containing proteins BRD4 and related BRD2/3 recognize histone lysine acetylations subsequently recruiting pTEFb, a CDK9/Cyclin-T kinase complex, to activate RNA polymerase II and target gene expression (109). Originally, these BRD genes were found aberrantly rearranged in malignant NUT midline carcinomas. A pioneering functional genomics screening of chromatin regulators in *MLL-*rearranged leukemia unveiled a role for BRD4 in maintenance of *C-MYC* expression and leukemia oncogenicity (110). Since advent of JQ1, the first highly selective and highly potent BRDi (showing a nano-molar range inhibition of BRDs) that competes BRD4 off acetylated histone ligands (111), multiple BRDis have been developed and their therapeutic effect seen in a wide range of human diseases including AML and other cancers (109). Mechanistically, BRDi such as JQ1 and I-BET151 repressed expression of a number of key oncogenic nodes including *C-MYC* and *BCL2* in mouse and human leukemia models carrying *MLL-*rearrangement (110, 112) (Figure 1F). BRDi was also found effective in treating non-*MLL-*rearranged AMLs such as those with *NPM1* mutation (113) or deletion of chromosome 7 and 7q [−7/del(7q)] (114), supporting their broader application in AML therapeutics. Even more potent BRDi, including a degrader derivative that can both inhibit BRD’s “reading” function and induce its proteasome-mediated degradation (115), have been developed, with several currently under clinical evaluation for the treatment of refractory AMLs (109). Following these encouraging advances, inhibitors of other RNA Pol-II activators such as the CDK7 and CDK9 kinases are on the horizon becoming a strategy to target transcriptional addiction to vital oncogenes seen in cancer (116, 117).

### Plant Homeodomain (PHD) Finger Inhibitors (PHDi)

The PHD finger-containing proteins comprise a large class of chromatin-associated proteins, some of which harbor the “reading” specificity toward H3K4 methylation (2, 106). In human AMLs, genes encoding the PHD finger-containing protein JARID1A (also known as KDM5A, a PHD finger-containing histone demethylase) and PHF23 were altered due to chromosomal abnormalities, resulting in in-frame fusion of their C-terminal H3K4me3-“reading” PHD finger to NUP98, a promiscuous gene translocation partner in human AMLs (32, 106). Despite generally low frequency of these genetic abnormalities in AMLs, the *NUP98-JARID1A/KDM5A* translocation was reported to be recurrent and detected in ~10% of the pediatric acute megakaryoblastic leukemia subtype (33). The produced NUP98-JARID1A or NUP98-PHF23 oncoproteins were highly potent in inducing AML transformation *in vitro*/vivo and rely on their H3K4me3-“reading” PHD finger domain to maintain high expression of “stemness” nodes, notably *HOX* and *MEIS1* (32, 118). Disulfiram, a previously FDA-approved drug, was found to carry the ability to inhibit binding of these PHD fingers to H3K4me3 possibly through structural alteration (119) and to selectively kill the leukemic cells transformed by *NUP98-PHF23* or *NUP98-JARID1A/KDM5A* (118) (Figure 1G). However, the potency and selectivity of disulfiram appear poor and the ligand-competitive inhibitors still remain to be developed for these PHD fusion oncoproteins.

## Targeting Chromatin “Erasers”

### HDAC Inhibitors (HDACi)

Histone deacetylases (HDACs) remove acetylation off histones to influent gene expression. HDACi (Table 1) including Vorinostat (also known as SAHA) and Panobinostat are the earliest inhibitors of epigenetic “erasers” approved by FDA for treatment of cutaneous T cell lymphoma and, recently, multiple myeloma. Currently, HDACi is under phase I/II trials of relapsed AML patients. As HDACs also deacetylate numerous non-histone substrates, effect of HDACi remains controversial as of the detailed mechanisms, especially through targeting histone versus non-histone proteins.

### LSD1 Inhibitors (LSD1i)

Lysine-specific demethylase 1 (LSD1/KDM1A) is the first identified histone demethylase with specificity toward H3K4 mono/di-methylation (H3K4me1/2) (120). Several LSD1i have been developed. In the *MLL-*rearranged leukemias, terminal differentiation arrest was partially enforced by LSD1, and LSD1i treatment induced myeloid differentiation and suppressed leukemogenesis *in vivo* (Figure 1H) (121). Mechanistically, LSD1i may perturb the H3K4me3/H3K4me2 ratio at MLL target genes thus reducing their transcription (121). Also, therapeutic effect of LSD1i was reported in AMLs without *PML-RARA* (i.e., non-APL AML), where LSD1i sensitized the pro-differentiation effect of ATRA, an agent only for *PML-RARA-*positive APLs (Figure 1H) (122). Here, combinational treatment of non-APL human AMLs with ATRA and LSD1i showed a potent anti-leukemic effect, with increased H3K4me2 and expression found at the myeloid differentiation genes (122). Several LSD1i are now in clinical trials in refractory AMLs.

### KDM4C Inhibitors

*KDM4C* (also known as *JMJD2C/GASC1*) encodes an “eraser” carrying the H3K9-demethylating and gene-activating activities. Like PRMT1, KDM4C was also found to interact with various AML oncoproteins including MLL-GAS7 and MOZ-TIF2 (103). Knockdown of *KDM4C* partially reversed target gene activation mediated by these AML fusion proteins. Moreover, pharmacological inhibition of KDM4C can be achieved by an inhibitor SD70 and proposed to be a potentially new AML treatment strategy (103) (Figure 1E).

## Perspectives

In short, epigenetic modulators emerge rapidly as potential drug targets for the treatment of currently incurable AMLs. With many showing high selectivity, high potency and/or promising drug-like properties, the already developed epigenetic inhibitors shall provide potential alternatives or adjuvants to current therapeutic arsenal that frequently relies on non-specific cytotoxic agents. While the area is in its infancy, we wish to pinpoint several directions that may broaden application of epigenetic inhibitors.

### Newly Validated Epigenetic Factors and Cancer Cell Dependency Pathways Remain to Be Targeted

An existing advance in understanding the biology of gene activation is recent identification of a YEATS family of protein domains as a new “reader” class of histone acylation such as acetylation and crotonylation (123, 124). In the *MLL-*rearranged AML cells, a YEATS domain harbored in ENL was recently shown to be crucial for tethering/stabilizing the MLL fusion proteins at sites with histone acetylation to induce downstream gene activation (125, 126). Similar mechanisms might be also functional among *DNMT3A-*mutated leukemias (Figure 1D, right) where *DNMT3A* mutations perturb efficient CpG methylation at cis-regulatory sites leading to elevated histone acetylation and increased binding of DOT1L-associated complexes that harbor YEATS-containing AF9 and ENL (96). Furthermore, LEDGF (lens epithelium-derived growth factor), a protein that mediates chromatin association of the MLL complex, was previously found to be essential for *MLL-*rearrangement-induced leukemic transformation (55). A recent work reports that the PWWP domain of LEDGF recognizes and “reads” H3K36 methylation added by the ASH1L methyltransferase at proximal promoter chromatin, and this event was found critical for recruiting/stabilizing MLL fusions onto target sites to activate gene expression in leukemia cells (127). Additionally, NSD1 and NSD3, two related H3K36 methyltransferases, were previously found to be aberrantly rearranged in ~15% of pediatric AMLs (31) and their “writing” SET domains represent the validated site that remains to be pharmacologically targeted (30). Thus, these discovered circuits should offer additional therapeutic opportunities, both in the “reading” domains (YEATS of AF9 or ENL; PWWP of LEDGF) and the catalytic “writing” domains (SET of ASH1L and NSD1/3), for AML treatment.

Identification of BRD4 as a novel AML dependency was achieved through shRNA-based functional screening of epigenetic factors (110). Small-guide RNA-based CRISPR/Cas9 technology has provided an alternative system to perform screening in human AML cell lines, which recently led to identification of the histone acetyltransferase KAT2A/GCN5 as an AML dependency gene (128). In future, functional genomics studies using a range of AML cell lines that represent various genetically defined AML subtypes, as well as validation with primary human AML samples, are likely to produce useful information for subtype-specific dependencies on epigenetic modulators, which would guide drug discovery efforts aiming to developing the personalized AML treatment.

### Implication in the Treatment of Pre-leukemic Disease

Somatic mutations of several epigenetic modulators (DNMT3A, TET2, IDH1/2) occur frequently among patients with pre-leukemia diseases such as MDS and apparently healthy individuals with clonal hematopoiesis or CHIP, an aging-related phenotype associated with increased risk of AML (21, 22, 25, 26). These mutations and resultant epigenetic deregulations are likely to be the “founder” lesion initiating pre-malignant disease and shaping subsequent malignant formation. Identification of the epigenetic vulnerabilities associated with these gene mutations in the context of AML shall provide useful information on how to treat premalignant diseases. For example, using a murine AML model harboring the coexisting kinase and *DNMT3A* mutations, a recent study demonstrated that *DNMT3A* mutation induced epigenetic dysregulation to promote “stemness” gene-expression programs, a process that can be reversed by DOT1L inhibitors (Figure 1D, right) (96). We speculate that the same mechanism/pathways act among premalignant diseases, and if so, the similar epigenetic inhibitors could reverse the premalignant alternations thus preventing malignant development in individuals with MDS or CHIP. In support, the epigenetic inhibitors and hypomethyating agents such as 5-Aza delay malignant transformation of MDS and are FDA-approved drugs for its treatment. However, as a life-threatening disease with a risk of conversion into AML, MDS has additional immediate needs to treat other complications such as anemia and transfusion associated iron overload, bleeding and infectious risk associated with the cytopenias. Currently, the definitive cure of MDS-associated leukemia risk is still allogeneic HSC transplantation. As for CHIP, there is consensus in the field that the relatively low risk of transformation of CHIP does not warrant the targeted therapies. Potential application of targeted epigenetic inhibitors in the treatment of pre-AML diseases such as MDS and myeloproliferative neoplasms warrants further investigation.

### Potential Drug Resistance and Combinational Therapy

Resistance to drug remains a challenge in achieving durable remissions in cancer and epigenetically targeted drugs are no exception. The molecular understanding of resistance in epigenetic therapy is just at its beginning. For example, *MLL-*rearranged leukemias with PRC2 loss, either pre-existing or acquired, are resistant to BRDi presumably due to enhanced transcription of oncogenes such as *MYC* (129); furthermore, recent reports documented acquisition of somatic mutation by blood cancer cells during resistance to BRDi or EZHi (129, 130). Conceptually, combinational treatment using two or more drugs that target multiple cancer cell dependencies should help overcome treatment resistance. Furthermore, regardless of drug resistance, combinational therapy should improve treatment when their potential toxic effect can be mitigated. As mentioned above, a good example is that LSD1i sensitizes non-APL AML cells to ATRA treatment (122). In addition, DOT1Li and BRDi are shown to be synergistic in treating *MLL*-rearranged leukemia, possibly due to functional collaboration between DOT1L and BRD4 at the highly transcribed super-enhancer genes (131). Future studies of drug resistance, toxicity, and combinational treatment strategies would be necessary to further develop and optimize the existing leads into those useful compounds for clinical trials.

## Author Contributions

RL and GW wrote the manuscript and generated the figures/tables.

## Conflict of Interest Statement

The authors declare that the research was conducted in the absence of any commercial or financial relationships that could be construed as a potential conflict of interest.
